# Nrf2 Induction Re-establishes a Proper Neuronal Differentiation Program in Friedreich’s Ataxia Neural Stem Cells

**DOI:** 10.3389/fncel.2019.00356

**Published:** 2019-07-31

**Authors:** Piergiorgio La Rosa, Marta Russo, Jessica D’Amico, Sara Petrillo, Katia Aquilano, Daniele Lettieri-Barbato, Riccardo Turchi, Enrico S. Bertini, Fiorella Piemonte

**Affiliations:** ^1^Unit of Neuromuscular and Neurodegenerative Diseases, IRCCS Bambino Gesù Children’s Hospital, Rome, Italy; ^2^Department of Biology, University of Rome Tor Vergata, Rome, Italy; ^3^IRCCS Fondazione Santa Lucia, Rome, Italy

**Keywords:** Nrf2, Friedreich’s Ataxia, neural stem cells, frataxin, neurogenesis, antioxidant, neurodegeneration

## Abstract

Frataxin deficiency is the pathogenic cause of Friedreich’s Ataxia, an autosomal recessive disease characterized by the increase of oxidative stress and production of free radicals in the cell. Although the onset of the pathology occurs in the second decade of life, cognitive differences and defects in brain structure and functional activation are observed in patients, suggesting developmental defects to take place during fetal neurogenesis. Here, we describe impairments in proliferation, stemness potential and differentiation in neural stem cells (NSCs) isolated from the embryonic cortex of the Frataxin Knockin/Knockout mouse, a disease animal model whose slow-evolving phenotype makes it suitable to study pre-symptomatic defects that may manifest before the clinical onset. We demonstrate that enhancing the expression and activity of the antioxidant response master regulator Nrf2 ameliorates the phenotypic defects observed in NSCs, re-establishing a proper differentiation program.

## Introduction

Friedreich’s Ataxia (FRDA) is an early-onset autosomal recessive disease with an incidence of 1:50000, caused by severely reduced levels of frataxin, a mitochondrial protein involved in iron–sulfur cluster synthesis, iron transfer, and antioxidant defense (Romeo et al., 1983; Dürr et al., 1996; Santos et al., 2010; Vaubel and Isaya, 2013). Although no evident signs of the pathology show up in the first 5–10 years of life, a subsequent development of movement coordination loss, cardiac hypertrophy, diabetes and progressive neurodegeneration occurs (Dürr et al., 1996; Folker et al., 2010; Weidemann et al., 2012), resulting in death at young age (Bürk, 2017). Cognitive differences in FRDA patients have also been assessed (Wollmann et al., 2002; Mantovan et al., 2006; De Nobrega et al., 2007; Corben et al., 2011, 2017; Nieto et al., 2013). Thus, even if the progressive degeneration of sensory neurons in the dorsal root ganglia (DRG) and in the dentate nucleus of the cerebellum are observed early upon pathology onset (Bürk, 2017), neuroimaging techniques revealed impairments in white/gray matter structure (Zalesky et al., 2014; Harding et al., 2016; Rezende et al., 2016) and in cerebral functional activation (Georgiou-Karistianis et al., 2012). Reports outlining these defects have been published since a decade (Selvadurai et al., 2018) and several lines of evidence suggest that frataxin deficiency could lead to their insurgence during fetal development (Cossée et al., 2000; Santos et al., 2001; Koeppen et al., 2017). However, studies on the pathogenic mechanism underlying FRDA during the neurogenesis are still lacking.

Recent reports show that a mouse model of the pathology, the Frataxin Knockin/Knockout (KIKO) mouse, manifests neurobehavioral defects on the 9th month of life that closely recapitulate the clinical human phenotype, including cerebellar ataxia, decreased peripheral sensitivity and motor strength and endurance impairments (Miranda et al., 2002; McMackin et al., 2017). Nevertheless, before the onset of the pathologic symptoms, mitochondrial and synaptic abnormalities are already present in the cerebellum (Lin et al., 2017a,b), suggesting that early pre-symptomatic defects may underlie the clinical onset and contribute to trigger the disease progression. In this context, the KIKO mouse model is a useful tool to search for earliest pathological changes, because it displays a slowly evolving phenotype, while biochemical and functional brain dysregulations arise earlier, thus closely recapitulating the clinical human phenotype (Lin et al., 2017a,b; McMackin et al., 2017; Cotticelli et al., 2019).

Oxidative stress and increased levels of free radicals play an important role in FRDA pathogenesis (Lupoli et al., 2018), where frataxin deficiency has been shown to reduce the expression of Nrf2 (the Nuclear factor erythroid-derived 2)-like 2 transcription factor NE2FL2 either in human FRDA fibroblasts (Paupe et al., 2009; Petrillo et al., 2017) and in mouse models of the disease (D’Oria et al., 2013; Shan et al., 2013; Anzovino et al., 2017; Dinkova-Kostova et al., 2018).

Nrf2 regulates the expression of several antioxidant enzymes and mounting evidences demonstrate an improvement of neurological phenotypes after induction of this signaling pathway (Dinkova-Kostova et al., 2018).

Redox signaling is critical in Nervous System development (Olguín-Albuerne and Morán, 2018) and Nrf2 has a relevant role in the neurogenic process, playing a key function in the regulation of neural stem cells (NSCs) features. In particular, its expression is directly correlated with NSCs proliferation and self-renewal, and its age-dependent down regulation determines the age-related NSCs loss of survival and function, impairing neurogenesis in subventricular zone of the lateral ventricles and in the dentate gyrus of the hyppocampus (Corenblum et al., 2016; Ray et al., 2018). Nrf2 activity and expression also play a role in the neuronal maturation process, as its overexpression and/or induced stabilization determine the increase of the mean length of neuron-differentiated neuroblastoma neuritis (Zhao et al., 2009), although the expression of the transcription factor is reported to constantly decrease throughout the differentiation process (Olguín-Albuerne and Morán, 2018). Importantly, the activation of Nrf2 signaling pathway is neuro-protective for progenitor cells exposed to amyloid beta (Aβ) deposits, a condition resembling the Alzheimer disease (Kärkkäinen et al., 2014), thus potentially representing an early therapeutic target in neurodegeneration.

Moving from these previous findings, in this study we analyzed the Nrf2 expression in NSCs isolated from the embryonic cortex of KIKO FRDA mouse and evaluated if an imbalance of Nrf2 signaling pathway may lead to early phenotypic defects in neurogenesis.

In addition, as two drugs, Sulforaphane (SFN) and EPI-743, are receiving increasing attention as promising candidates for the treatment of neurodegenerative diseases, including FRDA (Zesiewicz et al., 2018; Zhao et al., 2018), we analyzed the induction of Nrf2 pathway in response to those drugs in order to understand if an early activation of the transcription factor may trigger a neuro-regenerative mechanism in FRDA.

## Methods

### Ethics Statement

We conducted all mouse experimentations in accordance with accepted standard of humane animal care and with the approval by relevant local (Institutional Animal Care and Use Committee, University of Rome Tor Vergata) and national (Ministry of Welfare, license no. 324/2018-PR) committees. Experiments were carried out according to institutional safety procedures.

### NSCs Isolation, Culture, and Immunofluorescence Analysis

Neural stem cells were isolated from Frataxin KIKO C57/BL6 mouse (Charles River Laboratories International Inc., MA, United States) E13.5 (Bard et al., 1998) embryos as previously reported (La Rosa et al., 2016; Svetoni et al., 2017). Clonogenic assays were performed plating 5000 NSCs in 35-mm wells for each experimental point. After 5 days of culture, neurosphere number was counted and NPC clonogenicity expressed as the percentage ratio between plated cells and neurospheres formed. For differentiation assays, 20000 NPCs/well were plated on pre-coated poly-ornithine (Sigma-Aldrich, Saint Louis, MO, United States) and laminin-1 (Sigma-Aldrich) 4-well dishes. Cells were grown in NSCs medium, containing 1% v/v fetal bovine serum (FBS) (Gibco/Thermo-Fisher Scientific, United Kingdom) and incubated in a humidified atmosphere with 6% CO_2_, at 37°C, for 3 days. Immunofluorescence staining was performed after cell fixation in 4% (v/v) formaldehyde (Sigma-Aldrich) and permeabilization with 0.1% Triton X-100 in PBS, supplemented with 1% BSA. Samples were incubated with the mouse anti-TUBB3 (1:300, Sigma-Aldrich) primary antibody for 1 h at r.t. and with the FITC-conjugated (1:250) secondary antibody (Jackson ImmunoResearch, Cambridge, United Kingdom) for 1 h at r.t. Hoechst (Invitrogen, CA, United States) was added for 15 min, and fluorescence preserved using the Prolong Gold mounting solution (Invitrogen). 10 randomly fields were taken for each sample using a DMI6000B inverted microscope (Leica, Germany), equipped with a Pan-Neofluar 20X/0.75 objective lens. Data are represented as percentage of positive cells/total cells (evaluated by the number of total nuclei).

### RNA Isolation, RT-PCR, and RT-qPCR

Total RNA was extracted from NSCs using Total RNA purification kit (Norgen Biotek Corp., Canada), following manufacturer’s instructions. 1 μg RNA was retro-transcribed by M-MLV reverse transcriptase (Invitrogen) and used in quantitative RT-PCR (qPCR) experiments using Sybr green PCR master mix (Applied Biosystem, CA, United States) as described by manufacturer’s instructions. All primers used are reported in the table below. GAPDH gene expression was used to normalize qPCR experiments.

**Table:** 

Gene	**Forward primer (Fw)**	**Reverse primer (Rv)**
Nrf2	5′-TGAGAGGCAGCCATGACT-3′	5′-GTCCTTGTTTTCGGTATT-3′
NQO1	5′-CATCACAGGTGAGCTGAAG-3′	5′-CAGCTTCTTGTGTTCGGCCA-3′
HO-1	5′-TGACACCTGAGGTCAAGCAC-3′	5′-CTCTGACGAAGTGACGCCAT-3′
GAPDH	5′-CCTCGTCCCGTAGACAAAATG-3′	5′-TGAAGGGGTCGTTGATGGC-3′

### Immunoblotting

Neural stem cells were lysed in 50 mM Tris–HCl, pH 7.4, containing 100 mM NaCl, 1 mM MgCl_2_, 0.1 mM CaCl_2_, 1% NP-40, 0.5% sodium deoxycholate, 0.1% SDS and protease inhibitor cocktail (plus phosphatase and protease inhibitors) (Sigma-Aldrich). Proteins were separated by SDS–PAGE and transferred to polyvinylidene fluoride (PVDF) membranes (Amersham Biosciences Corp., United Kingdom). Antibodies were diluted in 0.1% Tween buffer (+ 5% BSA) as follows: rabbit anti-Nrf2 (1:500, Abcam, United Kingdom), mouse anti-NQO1 (1:2000, Novus Biologicals, United States), rabbit anti-HO-1 (1:2000, Abcam), mouse anti-Tubulin (1:1000, Sigma-Aldrich), rabbit anti-Frataxin (1:1000, Santa Cruz Biotechnology Inc., TX, United States). Signals were detected by enhanced chemiluminescence (ECL) (BioRad, CA, United States).

### Complex I Assay

Complex I (NADH:CoQ oxidoreductase, EC 1.6.5.3) activity was measured by following the absorbance decrease of NADH at 340 nm (ε = 6.81 mM^–1^⋅cm^–1^) in presence of the specific inhibitor rotenone (10 μM) (Carletti et al., 2014) and normalized for protein content.

### ROS Quantification

Three micromolar Dichlorofluorescin–diacetate (DCF–DA) (Sigma-Aldrich) was added to 96-well microplates (Greiner CELLSTAR^®^, Sigma-Aldrich) and incubated 1 h at 37°C in a humidified 5% CO_2._ Relative fluorescence units (RFU, λ_exc._ = 495 nm, λ_em._ = 530 nm), calculated by subtracting blank readings from all measurements, were taken using a plate spectrofluorometer (Enspire, Perkin Elmer). Results were normalized for cell number.

### Statistical Analysis

All data are expressed as mean ± SD. Student’s *t*-test was performed using Graphpad Prism software (RRID:SCR_002798).

## Results

### KIKO NSCs Show Proliferation, Clonogenicity, and Differentiation Defects

To analyze if neurodevelopmental defects may occur in FRDA, we isolated NSCs from 13.5 embryonic day of life (E) cortex of KIKO mouse, a well established FRDA animal model, which displays a slowly evolving phenotype despite early biochemical and functional brain deregulations (Lin et al., 2017a,b; Cotticelli et al., 2019), thus closely resembling patient’s pathologic progression (McMackin et al., 2017).

**FIGURE 1:**
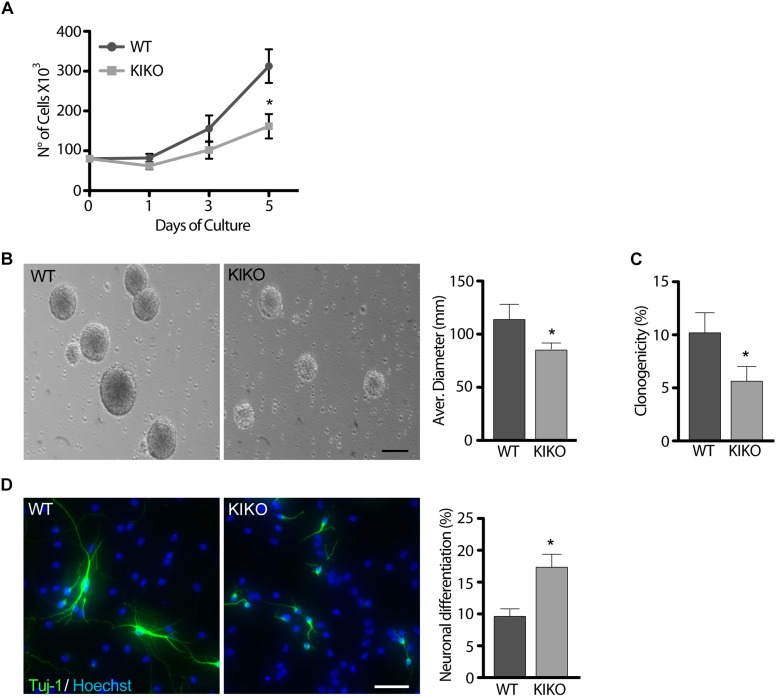
Frataxin depletion determines phenotypic defects in KIKO NSCs and neurons. **(A,B)** analysis of WT and KIKO NSCs proliferation assessed by growth curves experiments over 5 days **(A)**, and diameter evaluation on the fifth day **(B)**. **(C)** Clonogenic assay of WT and KIKO NSCs. Clonogenicity was expressed as the ratio between the observed neurospheres and plated NSCs. **(D)** Immunofluorescence analysis of the neuronal differentiation marker Tuj-1 in WT and KIKO NSCs cultured for 3 days in differentiating conditions. Graph on the right represents (mean ± SD) measurement of number of Tuj-1 positive cells, ^*^p < 0.05. Scale bars = 100 μm.

In culture, NSCs grow forming neurospheres that consist of a mix of stem and spontaneously differentiating cells (Conti et al., 2003; Galli et al., 2003). Growth curves over 5 days of culture showed a 48% reduction of KIKO NSCs proliferation, respect to WT NSCs (Figure 1A), and this was confirmed by 26% decrease of the average neurospheres’ diameter (Figure 1B). Furthermore, only ∼5% of KIKO NSCs were able to reform spheres upon disaggregation (Figure 1C), as assessed by analyses of the NSCs clonogenicity. As the reduced proliferation and clonogenicity of KIKO NSCs could be explained by an increase of spontaneous differentiation events, we further analyzed the KIKO NSCs differentiation index toward neuronal lineage (Figure 1D). After 3 days of differentiation, a 1.7-fold increase of neuronal differentiation was observed in KIKO NSCs, respect to WT NSCs, although an overall reduction of neuronal complexity was also evident (Figure 1D). These data highlight phenotypic defects in frataxin-deficient NSCs already at early stage of neurogenesis.

### Nrf2 Expression and Signaling Is Impaired in KIKO NSCs

**FIGURE 2:**
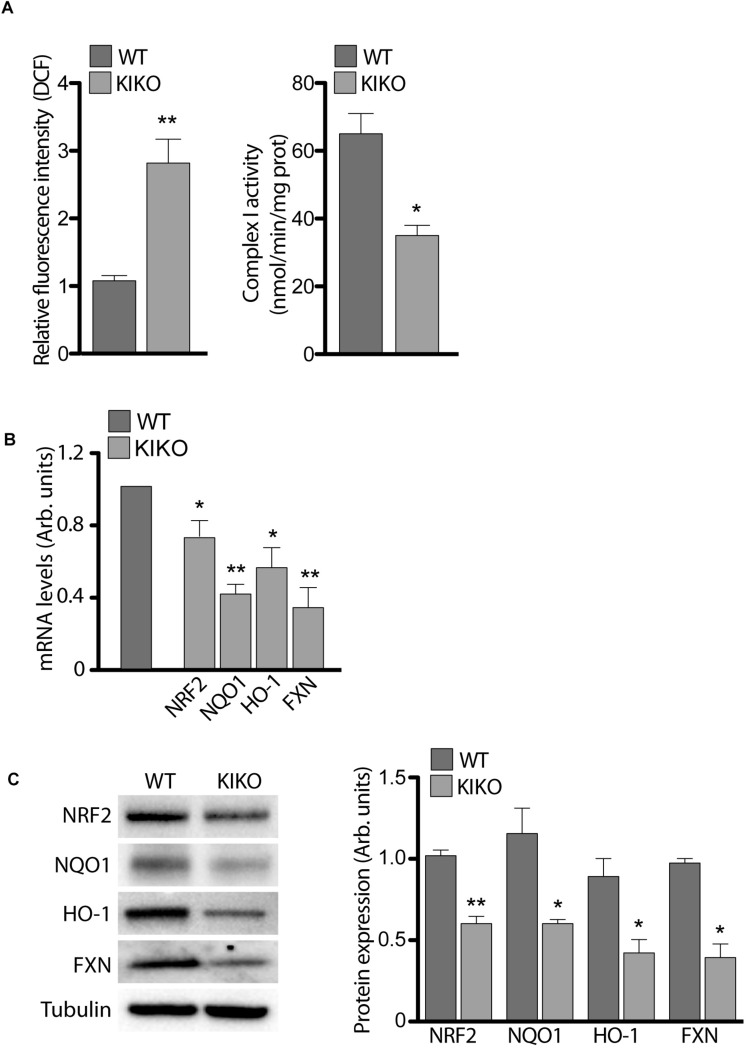
Frataxin depletion determines impairments in NRF-2 expression and signaling. **(A)** ROS determination (graph on the left) and Complex I activity (graph on the right), assessed in WT and KIKO NSCs. Cellular ROS were evaluated by measuring DCF fluorescence, normalized for cell number, while Complex I activity was expressed as nmol/min/mg prot. qPCR **(B)** and Western Blot analyses **(C)** and relative densitometric evaluation (graph on the right) of the expression of the transcription factor Nrf2, its targets (NQO1 and HO-1) and Frataxin, in WT and KIKO NSCs. GAPDH was used for qPCR normalization, while Tubulin was used as Western Blot loading control, ^*^p < 0.05 and ^∗∗^*p* < 0.01.

Given that the frataxin depletion causes ROS overload and iron-sulfur (Fe-S) cluster proteins impairment in FRDA (Lin et al., 2017b; Abeti et al., 2018; Lupoli et al., 2018), we measured the activity of mitochondrial (Fe-S) Complex I (CI) and ROS levels in KIKO NSCs, in order to validate our model. As shown in Figure 2, CI activity was significantly decreased (46%) in KIKO NSCs, whereas ROS increased 3-times respect to WT NSCs, thus confirming the molecular key features of the disease.

As several studies show Nrf2 impairment in post-natal tissues of FRDA patients and in frataxin-deficient cells (Paupe et al., 2009; D’Oria et al., 2013; Shan et al., 2013; Petrillo et al., 2017), we analyzed Nrf2 expression in KIKO and WT NSCs, in order to evidence a potential involvement of the transcription factor in the defects described above. As reported in Figure 2, Nrf2 was reduced in KIKO NSCs either as mRNA (20% decrease, A) and as protein level (40% decrease, B). In the same way, a significant decrease of two representative Nrf2 target genes was detected in KIKO NSCs, compared to WT NSCs. In particular, NADPH Quinone Oxidoreductase 1 (NQO1) was 60% reduced as mRNA (Figure 2A) and 50% as protein amount (Figure 2B), and Heme Oxigenase-1 (HO-1) showed a 50% decrease both as mRNA (Figure 2A) and protein level (Figure 2B). As expected, a 60% decrease of frataxin expression (mRNA and protein) was also detected in KIKO NSCs (Figures 2A,B).

These findings confirm previous studies showing a frataxin-mediated Nrf2 deficiency in cell and mouse models of FRDA (Paupe et al., 2009; D’Oria et al., 2013; Piermarini et al., 2016; Anzovino et al., 2017; Petrillo et al., 2017) but, additionally, they represent a progress in understanding the pathogenesis of FRDA because, for the first time, an early impairment of Nrf2 signaling is described already during neurogenesis. Furthermore, given the role of Nrf2 in the neurogenic process (Zhao et al., 2009; Corenblum et al., 2016; Olguín-Albuerne and Morán, 2018; Ray et al., 2018), the defective Nrf2 pathway may also underlie the loss of stemness potential and the increased cell differentiation toward the neuronal lineage evidenced in KIKO NSCs (Figure 1).

### SFN and EPI-743 Treatments Restore Nrf2 and Nrf2-Target Gene Expression

Nrf2 inducers have been demonstrated to promote the activation of Nrf2/ARE signaling in frataxin silenced motor neurons (Piermarini et al., 2016; Petrillo et al., 2017). Thus, in order to evaluate the effect of Nrf2 activation on the KIKO NSCs defects, we treated KIKO NSCs with the classical Nrf2 inducer SFN and with EPI-743, a para-benzoquinone developed for the treatment of mitochondrial diseases (Enns et al., 2012; Martinelli et al., 2012; Zesiewicz et al., 2018).

**FIGURE 3:**
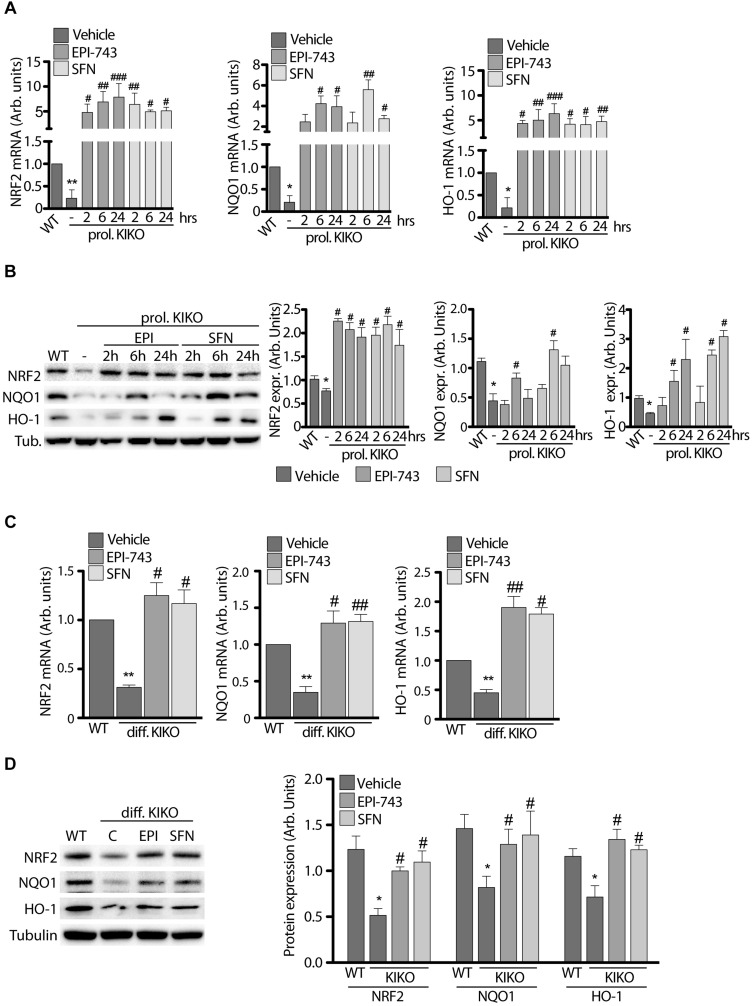
Antioxidant treatment increases NRF2 and downstream gene expression in proliferating and differentiated NSCs. **(A)** qPCR and **(B)** Western Blot analysis with relative densitometric quantification (**B** graphs on the right) of NRF2, NQO1 and HO-1 expression in WT and KIKO NSCs cultured in proliferating conditions and treated for 2, 6, and 24 h with 1 μM EPI-743 or 5 μM SFN. qPCR analyses **(C)** and Western Blot experiments **(D)** of NRF2, NQO1, and HO-1 mRNA and protein expression levels in differentiated WT and KIKO NSCs treated or not with 1 μM EPI-743 or 5 μM SFN during the differentiation protocol. GAPDH was used for qPCR normalization, Tubulin was used as Western Blot loading control. ^*^p < 0.05 and ^∗∗^*p* < 0.01 vs. WT. ^#^*p* < 0.05, ^##^*p* < 0.01, and ^###^*p* < 0.001 vs. vehicle-treated KIKO.

qRT-PCR and western blot analyses were performed either under conditions of KIKO NSCs proliferation (Figures 3A,B) and following neuronal differentiation (Figures 3C,D).

As shown in Figure 3, both compounds significantly induce the expression and stability of Nrf2 in proliferating KIKO NSCs, compared to untreated KIKO NSCs, showing a consistent increase of mRNA (Figure 3A) and protein amount (Figure 3B) already after 2 h treatments (10-fold increase mRNA and 4-fold increase protein level), remaining high throughout 24 h. Also Nrf2 target genes were significantly induced after EPI and SFN treatments, with NQO1 reaching a peak at 6 h drugs (3-fold increase protein amount with EPI and 6-fold increase with SFN), whereas HO-1 showed a growing increase over time (4-fold protein increase with EPI and 6-fold with SFN) (Figures 3A,B).

A significant induction of Nrf2 and its down-stream genes was also found after 3 days neuronal differentiation of KIKO NSCs (Figures 3C,D), with 1.7- and 2.2-fold increases of protein level, following respectively EPI and SFN 24 h treatments. Similarly, Nrf2 target genes were induced with EPI and SFN both as mRNA (3.6-fold increase, Figure 3C) and protein level (1.9-fold increase, Figure 3D).

Overall, these findings highlight the effectiveness of the drug-mediated Nrf2 induction in restablishing the antioxidant defense signaling in KIKO NSCs, thus leading to suggest the transcription factor as a potential early target of therapy.

### SFN and EPI-743 Revert Phenotypic Defects in KIKO NSCs and Promote Neuronal Complexity and Differentiation

**FIGURE 4:**
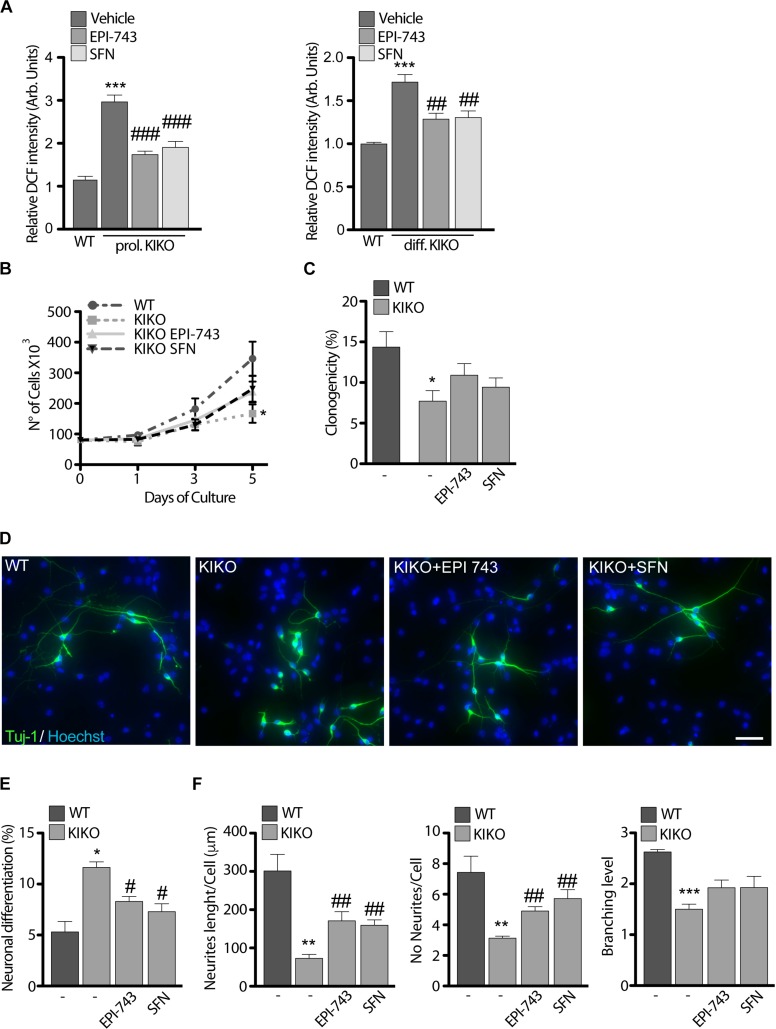
Antioxidant treatment partially re-establishes KIKO NSCs differentiation program toward neuronal lineage. **(A)** ROS determination in WT and KIKO NSCs treated with 1 μM EPI-743 or 5 μM SFN or vehicle in proliferating condition (left graph) or after differentiation (right graph). The fluorescence produced by the oxidation of DCF was normalized for cell number. **(B)** Analysis of WT and KIKO NSCs proliferation, assessed by growth curve over 1, 3, and 5 days of culture in proliferating condition and after treatment with 1 μM EPI-743 or 5 μM SFN. **(C)** Clonogenic assay of WT and KIKO NSCs cultured in proliferating condition and treated with 1 μM EPI-743 or 5 μM SFN or vehicle. Clonogenicity was expressed as the ratio between counted neurospheres and plated cells. **(D)** Representative images of immunofluorescence assay to evaluate NSCs differentiation toward neuronal lineage (Tuj-1 positive cells) in WT and KIKO NSCs treated with vehicle, 1 μM EPI-743 or 5 μM SFN. The graph in panel **(E)** represents measurements of Tuj-1 positive cells number (mean ± SD). **(F)** Analysis of WT and KIKO neuronal complexity, assessed by evaluating average neurites’ length (left), average neurites’ number (center), and average branching level (right) in samples treated or not with 1 μM EPI-743 or 5 μM SFN along the differentiation protocol. ^*^p < 0.05, ^∗∗^*p* < 0.01, and ^∗∗∗^*p* < 0.001 vs. WT. #*p* < 0.05, ##*p* < 0.01, and ###*p* < 0.001 vs. vehicle-treated KIKO. Scale bars = 100 μm.

Following the drug-mediated rescue of Nrf2 function, we evaluated the effect of EPI-743 and SFN on KIKO NSCs ROS production (Figure 2A). Both treatments consistently reduced ROS overload, either in proliferating condition or during the differentiation process, thus re-balancing the cellular redox environment. Prompted by these results and by previous studies showing that the Nrf2 activation restored neurites’ network and axonal re-growth in FRDA silenced neurons (Piermarini et al., 2016; Petrillo et al., 2017), we asked if Nrf2 induction was able to rescue the phenotypic defects observed in KIKO NSCs. As evidenced by growth curves (Figure 4A) and clonogenic assays (Figure 4B), both SFN and EPI-743 treatments trigger a positive effect on proliferation (1.7-fold increase) and stemness potential (1.4-fold increase) in KIKO NSCs culture, compared to untreated KIKO NSCs, although this rise was not enough to reach the statistical significance.

Moving from the data reported in Figure 1C, showing a consistent increase of spontaneous differentiation events in KIKO NSCs accompanied by a reduction of neuronal complexity, we further tested the efficacy of SFN and EPI-743 on KIKO NSCs neuronal morphology and differentiation rate (Figure 4C).

When chronically administrated, EPI-743 and SFN re-established a proper differentiation index in KIKO NSCs, leading to a 28 and 30% decrease of differentiating events, respectively (Figures 4D,E). A re-organization of neurites’ network was also evidenced following treatments, with a significant increase of neurites’ length (2.4-fold increase with EPI and 2.2-fold increase with SFN) and neurites’ number (1.6-fold after EPI and 1.8-fold after SFN) (Figure 4F).

These findings show that the drug-mediated Nrf2 activation contributes to a partial recovery of the neuronal morphology and differentiation process in KIKO NSCs. Thus, also based on the evidence of the pre-symptomatic Nrf2 impairment in KIKO mouse model, we believe that this study paves the way for Nrf2 as an early drug target for FRDA.

## Discussion

Although FRDA clinical symptoms manifest between the first and the second decade of life, patients are exposed to frataxin deficiency since development (Bürk, 2017), thus pre-symptomatic defects may contribute to determine the onset and the worsening of FRDA phenotype (Cossée et al., 2000; Santos et al., 2001; Georgiou-Karistianis et al., 2012; Rezende et al., 2016; Selvadurai et al., 2018). Based on this assumption, the evaluation of early pathological changes may be essential to understand the pathogenesis of the disease and to identify new targets for innovative early therapies.

In most cases, indeed, brain samples used for analysis are available from late-stage individuals, thus evidences of early pathological changes can be lost during the disease progression. In this regard, KIKO FRDA mice represent a very useful model to analyze defects in the pre-symptomatic stage of the pathology, because they display a slowly evolving phenotype such as in patients’ disease progression, while biochemical and functional brain defects arise earlier (Lin et al., 2017a,b; McMackin et al., 2017; Cotticelli et al., 2019). Furthermore, unlike the lethal prenatally models in which frataxin is completely depleted and the neuron-specific knockouts showing a too severe early onset phenotype (Cossée et al., 2000; Simon et al., 2004), KIKO mice display frataxin levels close to patients’ values (20–30% of control levels) (Sahdeo et al., 2014; Lazaropoulos et al., 2015), and neurological signs (i.e., cerebellar gait ataxia, decreased peripheral sensitivity, and motor strength impairment) resembling those occurring in late-onset FRDA patients (McMackin et al., 2017). These neuro-pathological symptoms arise upon the 9th month of life in the KIKO mouse (McMackin et al., 2017), while the deregulation of cerebellar synaptic circuits (Lin et al., 2017b) and mitochondrial impairments (Lin et al., 2017a) occur already at asymptomatic ages of 1st and 3rd months, respectively. Therefore, as reported for other neurodegenerative diseases (Shirendeb et al., 2012; Cai and Tammineni, 2016), we hypothesize that early dysfunctions may be responsible for the onset of FRDA and contribute to address the pathological evolution of the disease.

In light of this, we analyzed NSCs isolated from the cortex of 13.5 days embryonal life (E) of KIKO mice, in order to highlight weather defects were present already during neurogenesis. We investigated the morphological and biochemical phenotype of KIKO NSCs and their proliferative and stemness potential.

Our findings show proliferation and clonogenic defects, premature neuronal differentiation and loss of neuronal complexity in E13.5 KIKO NSCs (Figures 1A–D), thus suggesting that frataxin deficiency could induce defects already during neurodevelopment in FRDA and potentially lead to impairments in the white/gray matter structure and connectivity observed in patients (Georgiou-Karistianis et al., 2012; Zalesky et al., 2014; Harding et al., 2016; Rezende et al., 2016).

A tight control of NSCs proliferation, stemness potential and differentiation is critical for a proper brain development (Sun and Hevner, 2014; Taverna et al., 2014), and defects perturbing this balance can lead to the premature exhaustion of stem cells pool, determining the reduction of cortical thickness (Sun and Hevner, 2014; La Rosa et al., 2016). In line with this, two recent studies show thickness and volumetric reduction of cortical lobes in FRDA patients (Rezende et al., 2016; Selvadurai et al., 2016), thus supporting our hypothesis according to which the defects we observed in “*in vitro*” KIKO NSC could resemble the impairments that determine alterations in patients.

Neural stem cells strictly depend on low oxidative environment to maintain their stemness capability (Khacho et al., 2019), and the switch between glycolytic and oxidative metabolism determines an increase of oxidative species that drives the differentiation process (Tormos et al., 2011; Khacho et al., 2016; Zhou et al., 2016). As frataxin deficiency has been reported to enhance production of cellular free radicals in patients and in KIKO cells (Abeti et al., 2018) and Nrf2 deficiency has been described in post-natal FRDA tissues and in frataxin-silenced motor neurons (D’Oria et al., 2013; Piermarini et al., 2016; Petrillo et al., 2017), we evaluated if KIKO NSCs exhibit Nrf2 impairment during neurogenesis. Importantly, the expression of Nrf2 and two target genes (HO-1 and NQO1) is down regulated in KIKO NSCs, respect to the WT NSCs (Figures 1E,F), evidencing a defective antioxidant response in FRDA already at early stages of the disease.

Nrf2 is a key factor in neurogenesis regulation, and redox signaling is crucial in nervous system development (Zhao et al., 2009; Kärkkäinen et al., 2014; Olguín-Albuerne and Morán, 2018). Thus, the decrease of Nrf2 levels we detected in KIKO NSCs could be responsible for the reduction of their proliferation and stemness potential, allowing an anticipated differentiation program to take place (Figures 1A–C). Notably, it has been previously reported that in the neurogenic niches of the adult brain, the progressive reduction of Nrf2 expression in the stem cell pool correlated with the age-dependent decline of neural progenitors, whereas its overexpression improved NSCs proliferation and regeneration (Zhao et al., 2009; Corenblum et al., 2016). Therefore, the deregulation of Nrf2 expression, evidenced in KIKO NSCs, may underlie the loss of stemness potential and the increased cell differentiation toward the neuronal lineage. Moreover, as in frataxin-silenced neurons the Nrf2-mediated redox imbalance leads to structural impairments and axonal degeneration (Petrillo et al., 2017), we believe that the decrease of Nrf2 expression in KIKO NSCs may also be responsible for defects in the neuronal maturation and in the reduced neuronal complexity (Figure 1D). This reduced Nrf2 expression could contribute to explain the recent hypothesis by which the DRG of FRDA patients undergo an early neuronal hypoplasia participating to the late pathologic neuro-degenerative process (Koeppen et al., 2017).

Finally, as no effective therapies have been currently approved for FRDA and the Nrf2 activation was neuroprotective in models of Parkinson’s disease and in multiple sclerosis (Benarroch, 2017), we treated KIKO NSCs with two Nrf2 inducers (SFN and EPI-743), known to be effective in frataxin-silenced motor neurons (Piermarini et al., 2016; Petrillo et al., 2017) and in chronic neurodegenerative diseases (Martinelli et al., 2012; Sadun et al., 2012; Chicani et al., 2013; Tarozzi et al., 2013; Sun et al., 2017; Zhang et al., 2017; Hou et al., 2018; Morroni et al., 2018; Panjwani et al., 2018; Zesiewicz et al., 2018; Zhao et al., 2018).

Both SFN and EPI-743 treatments partially restore proliferation and clonogenicity of KIKO NSCs, although physiological levels were not fully reached (Figures 4A,B). Technical limitations in NSCs culture conditions could explain this partial result. NSCs grow as cellular aggregates and, as the growth of the sphere increases, this makes difficult for compounds reaching cells residing inside the spheres. Thus, it is possible that the drugs’ effect on proliferation and clonogenic potential occurs in the first days of culture, but becomes less effective as the culture grows. Nevertheless, when SFN and EPI-743 were administrated on spread-cultured differentiating NSCs, a significant rescue of the KIKO NSCs defective phenotype was observed (Figures 4C–E), demonstrating that a balanced Nrf2 signaling axis is required so that a proper differentiation process takes place.

Overall, our study highlights two main findings: (1) the Nrf2 signaling pathway is impaired in the pre-clinical KIKO NSCs model; (2) the reduced expression of frataxin leads to phenotypic defects that are partially restored upon drug-driven Nrf2 induction. These findings, besides confirming pathological hallmarks in KIKO NSCs, provide evidences of up-stream neurogenesis defects occurring in FRDA.

It is also important to note that the premature exhaustion of NSCs pool during fetal neurogenesis, due to reduced proliferation and self-renewal together with the increase of neuronal differentiation, may contribute to defects in cortical thickness (La Rosa et al., 2016), thus potentially determining cerebral and cerebellar abnormalities reported in FRDA patients (Selvadurai et al., 2016, 2018). Future studies are needed to “*in vivo*” validate our findings on brain tissues obtained from post-natal KIKO mice, in order to evaluate if neurogenesis deficits may impact on clinical symptoms. This should be of paramount importance for early intervention possibly targeted to Nrf2 activation, taking advantage of highly feasible and tolerable treatments.

## Data Availability

Data presented in the manuscript are available from the corresponding author upon request.

## Author Contributions

PL performed and supervised all the experiments, and analyzed the data. MR cultured the NSCs and performed the clonogenic, growth, and differentiation assays, and western blot analysis. JD’A performed the ROS quantification and Complex I activity assay. SP carried out the qRT-PCR measurements. PL and RT managed the KIKO mice and isolated the NSCs from embryos. KA, DL-B, and EB revised the manuscript. PL and FP designed the experiments, and drafted and edited the manuscript.

## Conflict of Interest Statement

The authors declare that the research was conducted in the absence of any commercial or financial relationships that could be construed as a potential conflict of interest.
